# Assessing progress in gender-transformative sexual and reproductive health interventions: how does practice-based knowledge compare with published evidence in sub-Saharan Africa?

**DOI:** 10.1080/16549716.2026.2685464

**Published:** 2026-06-23

**Authors:** T. K. Sundari Ravindran, Asha S. George, Tanya Jacobs, Kefilath Bello, Woldekidan Amde

**Affiliations:** a Independent Researcher, Trivandrum, India; b School of Public Health, University of the Western Cape, Cape Town, South Africa; c Centre de Recherche en Reproduction Humaine et en Démographie, Cotonou, Bénin

**Keywords:** Feminist evaluation, gender-responsive evaluation, gender power relations, gender-responsive approaches, SDG 5: Gender equality

## Abstract

**Background:**

Discriminatory gender norms and unequal power relations shape sexual and reproductive health (SRH) outcomes in sub-Saharan Africa. Yet, approaches to assessing progress in gender-transformative interventions remain underdeveloped.

**Objective:**

This paper compares measures and approaches to gender-transformative SRH interventions in the published evidence with practitioners’ perspectives in sub-Saharan Africa.

**Methods:**

The study draws on two data sources from a broader research initiative: in-depth interviews with 19 African practitioners and researchers engaged in gender-transformative health programming, and 44 published evaluations of 20 gender-transformative SRH interventions implemented between 2012 and 2025. We examine what is assessed, how assessments are designed and implemented, and when they are conducted.

**Results:**

Practitioners emphasised assessments that capture multi-level, context-specific changes; participatory processes; longitudinal, qualitative methods; and documentation of change pathways and sustainability. In contrast, published studies predominantly measured short-term, individual-level attitudinal and behavioural change – especially among men – using standardised, externally defined instruments reliant on self-reports, with limited attention to women’s empowerment, intersectional inequalities, institutional or health-system change, or long-term sustainability.

**Conclusions:**

Findings reveal significant misalignments between practice-based knowledge, feminist and gender-responsive evaluation principles, and published evaluation studies. To strengthen investment and learning, we propose advancing assessment frameworks that: engage with communities in the design, implementation, and assessment of gender-transformative SRH interventions; use gendered health outcome indicators; use participatory approaches; document processes and unintended effects; validate men’s self-reports with other perspectives; and extend evaluation beyond project cycles. Aligning evidence generation with grounded African practice is essential for more effective and sustainable gender-transformative SRH programming in sub-Saharan Africa.

## Paper Context

- **Main findings:** The measures and processes used by most of the reviewed intervention studies to assess gender-transformative changes in SRMH programs in sub-Saharan Africa appear to be neither fully aligned with practice-based perspectives from the region nor with the broader literature on principles for measurement and assessment in gender-transformative programs.
- **Added knowledge:** The measurement approach of practitioners and researchers was more aligned with the principles for evaluating gender-transformative programs, such as examining changes at multiple levels, using participatory processes to develop context-specific measures and implement assessments, and documenting the sustainability of changes.
- **Global health impact for policy and action:** The research calls for concerted effort and investment to advance nuanced approaches to assessing the success of gender-transformative interventions, drawing on the strengths of practice-based knowledge and rigorous intervention studies.

## Background

Substantial evidence exists to indicate that discriminatory gender norms and roles, unequal access to and control over resources, and decision-making power profoundly impact health-seeking and health outcomes, especially in women and girls, but also harm men and boys through rigid expectations about masculinity [1–3].

Gender-transformative health interventions aim to achieve positive health outcomes by challenging unequal gender norms and other gender-based inequalities at the individual, interpersonal, and structural levels, whereas gender-specific health interventions address sex- and gender-specific health needs of all individuals to improve health outcomes [4].

Although gender-transformative interventions in health have been implemented for more than two decades, particularly in sexual and reproductive health [5], reviews of SRH interventions in LMICs have highlighted the lack of appropriate measures and assessment tools, which limit the value of evaluation studies in assessing the effectiveness of gender-transformative interventions in health [6–8].

The need to further strengthen commitments to gender equality and sexual and reproductive health, grounded in robust evidence, is vital in African contexts, where gender equality, beyond being an important development outcome, is key to achieving other development outcomes [9]. Until the latest funding cuts, which risk undoing gains thus far, several multilateral and bilateral agencies contributed substantial funding to gender-transformative programming across various sectors, including health. Furthermore, several studies on gender-transformative SRH interventions in the region have been published over the last two decades [10]. However, thought leadership has primarily originated in high-income countries [10,11]. A recent study highlights that research leadership in sub-Saharan Africa is weakened by interconnected barriers at the individual, institutional, and structural levels, contributing to the predominance of high-income country authorship in research on gender-transformative sexual and reproductive health programming [11]. Gender power relations are inextricably rooted in the local contexts. Limited local ownership and leadership in SRH research examining gender-transformative changes risk inadequately capturing local realities. Drawing on the practice-based knowledge of practitioners implementing gender-transformative interventions in sub-Saharan Africa would help address this limitation.

Practice-based knowledge is ‘*the cumulative knowledge and learning acquired by practitioners through years of innovation, reflection, and refinement. It includes insights gained from observations, conversations, direct experience, and programme monitoring’* [12].

This paper compares the measures and approaches to gender-transformative SRH interventions in the published evidence with the perspectives of practitioners in sub-Saharan Africa. The intention is to stimulate reflection on how intervention studies that provide the ‘evidence base’ to donors and policymakers for what constitutes ‘best buys’ could be better aligned with the practice-based knowledge and insights of the region’s practitioners and researchers, and on how we can draw on the strengths of both these approaches to strengthen the evidence base overall.

Taking stock of the what, how, and when of assessments of gender-transformative interventions, from the perspective of practitioners and of researchers, and drawing on their combined strengths, would contribute to more effective programming and meaningful investments that better support and strengthen gender equality and justice across sub-Saharan Africa. Such an exercise is critical at a time when there is a pushback on gender equality by anti-gender campaigns funded by local and international sources [13].

The present paper engages with the following questions:

- According to the practice-based knowledge of sub-Saharan African researchers and practitioners, what are robust approaches to assessing gender-transformative sexual and reproductive health (SRH) interventions, and what are the challenges in operationalising them?
- What is the published evidence on assessment approaches adopted by gender-transformative SRH interventions in Africa?
- How well aligned are these two sets of evidence, and what are the implications of the alignment or lack of it for gender-transformative SRH interventions in sub-Saharan Africa?

## Methods

### Data sources

This paper draws selectively on two sources of information to examine the assessed outcomes, the processes and methodological approaches used, and the timing of the assessments of gender-transformative sexual and reproductive health interventions across sub-Saharan Africa. Both sources of information were part of the same research initiative on gender-transformative SRH interventions and included information on assessments and evaluations of these interventions. However, the two studies were independent, and the data available from them do not exhibit one-to-one congruence across all aspects examined in this paper.

#### Exploratory qualitative study

The first source is an exploratory qualitative study of African perspectives on gender-transformative health interventions based on in-depth interviews with African practitioners and researchers. Participants were recruited from an initial pool of approximately 40 individuals identified through networks and communities of practice in the field across the African continent, and recommended by practitioners and researchers working to advance gender equity in health. Following a review of profiles, 25 respondents were invited to participate, chosen for intersectional (including male, LGBTQIA+, and disability), generational, regional, linguistic, and sectoral representation. Of those invited, 19 were available and agreed to take part in the interviews. The interviews were conducted virtually via Zoom from January to May 2023. Each author conducted three to four interviews. Four interviews were conducted in French, while the remaining 15 were done in English.

The interviews sought to understand how the participants have operationalised the concepts of gender-transformative and gender-responsive approaches, their perspectives on approaches to assessing the progress of gender-transformative interventions, their views on achievements and challenges experienced, and insights and suggestions on what constitutes critical actions and principles to implement gender-transformative health programs in the African settings/contexts.

The interviews were audio-recorded, and the interviewers carefully cross-checked the Zoom transcriptions against the audio recordings, correcting and finalising the transcripts. A two-step coding process was adopted. In the first step, the research team read the transcripts and organised the information into broad categories based on the key areas covered in the interviews. The second step involved reading and rereading the information and applying open-style inductive coding within each category [14]. Coding was done manually, and the codes were grouped into themes around which information was summarised. The research team kept a memo of participant quotes from the transcripts and excerpts from the field notes, which were used to illustrate the findings reported [15]. For this paper, we used only data pertaining to their perspectives and practices regarding the assessment of gender-transformative SRH interventions.

#### Published evaluation studies

The second source comprises 44 evaluation studies of 20 gender-transformative SRH interventions in sub-Saharan Africa. Of these, 27 evaluation studies were drawn from a larger scoping review of 52 publications identified in PubMed and Scopus between 1 January 2012, and 12 December 2022 [10]. The larger scoping review included publications that evaluated gender-transformative interventions or focused on the gender dimensions of sexual and reproductive health interventions in sub-Saharan Africa. Details of the screening and extraction process for the scoping review have been described in detail elsewhere [10]. The 27 articles included in the present study were selected from the 52 scoping review articles that met the criterion of being evaluation studies of gender-transformative SRH interventions.

This selection was supplemented with articles identified through a manual search of Google Scholar, and the 20 distinct interventions referenced in the 27 articles were evaluated. The intention was to capture the full range of available evidence on these interventions. This supplementary search initially identified 50 additional articles related to at least one of the 20 interventions. Following screening, we selected 17 of the 50 additional publications for full-text review, all of which were evaluation studies of the same 20 interventions identified in the original 27 studies. This process brought the total number of reviewed published intervention studies to 44.

#### Analysis

Data were extracted from the 44 evaluation studies on objectives, health areas, target groups, duration, activity types, outcomes assessed, methodologies, timing, and evaluation results. Results were consolidated and presented by intervention rather than by individual article to more clearly capture the nature of assessments conducted over time, including the sequencing of studies and the evolution of findings.

The analysis consisted of comparing data extracted from the two sources on

- What were (or should be) the outcomes assessed?
- How were (or should) the evaluations/assessments (be) carried out, both in terms of the process through which outcomes for assessment were identified, and in terms of the methodologies adopted
- When and how often during the life course of the interventions were the evaluations/assessments carried out (or should be carried out)?

The data available for each of these questions was uneven across the two sources. The first source included information not only on what had been done but also on participants’ perspectives on what ought to be, while the second source, being published information, describes actual practice. Additionally, not all participants responded in detail across all three areas of exploration mentioned above. Nevertheless, the comparison yielded essential insights.

#### The research team

We are a cross-generational team of five public health researchers (SR, AG, TJ, KB, WA) from the Global South, of which three (TJ, KB, and WA) are from sub-Saharan Africa, and two (SR and AG) are from India. Two team members (SR and TJ) have extensive experience in grassroots engagement with gender-transformative SRH interventions. Our social locations and experiences have helped us understand the issues this study explores.

#### Ethics approval

The research project is approved by the Biomedical Research Ethics Committee of the University of the Western Cape (BM22/9/2), South Africa. The research was conducted in accordance with the principles of the Declaration of Helsinki.

## Results

In this section, following the presentation of sample characteristics, we present perspectives from practitioners and researchers (referred to from now on as participants) and juxtapose them against what we found from the scoping review about the following aspects of assessment or measurement in gender-transformative SRH interventions:

What outcomes are to be assessed?
How (the processes of deciding on the assessment outcomes and approaches, and the study designs and methods used)
When (in the life cycle of an intervention, how often)

### Sample characteristics

#### Qualitative study

The 19 participants were from Burkina Faso (1), Ghana (2), Niger (2), Ethiopia (2), Kenya (3), Rwanda (2), Uganda (2), Malawi (1), and South Africa (4). With respect to organisational affiliation, participants represent a range of sectors: nine were from civil society organisations (including one working with an international NGO), seven were from universities and research groups (including two affiliated with international research institutions), two from United Nations agencies, and one from a donor agency. The majority of participants (13) were mid-career professionals with substantial programmatic or academic/research experience, while the remaining six were at more advanced stages of their careers. Within their respective organisations, respondents were engaged in a broad set of interrelated areas, including work related to sexual and reproductive health and rights (SRHR); protection and promotion of rights; and capacity development within their own or partner institutions. Participants also represent interdisciplinary backgrounds, including medicine, public health, sociology, anthropology, and related fields.

While deliberate efforts were made to capture a diverse range of experiences across gender and health, geographic contexts, and institutional types, participants were not directly asked to disclose their social identity characteristics. Nevertheless, based on available information, participants appear to represent diverse gender identities, abilities, and racial backgrounds [15].

#### Published evaluation studies

Table 1 provides an overview of the 44 published studies (16–59) that evaluate 20 gender-transformative health interventions included in the analysis. The table documents the objectives, health areas, target groups, duration, and timing of the intervention; the methods of evaluation or assessment; the outcomes assessed; and the evaluation findings on whether the intervention was successful, i.e. achieved its objectives.

**Table 1.** A description of the gender-transformative sexual, reproductive, and maternal health interventions analysed.

S. No	Reference numbers	Objectives and nature of intervention	Health area	Intervention target groups	Duration of the entire intervention	Evaluation methods and timing	Outcomes assessed	Evaluation findings
1	[16,17]	**FOWODE GENDER BUDGET INITIATIVE, KABALE, UGANDA. Two publications: Bamanyaki (2015) and Bamanyaki and Holvoet (2016)**						
		Objective: To bring about gender-responsive maternal health service delivery and increase the knowledge and demand for skilled maternal health care, and its utilisation through local-level civil society-led gender-responsive budgeting (GRB) initiatives Interventions: Gender and GRB training provided to female district councilors and technocrats; technical support provided to technocrats; mobilisation and sensitisation of grassroots citizens’ groups on gender, rights, and planning and budget processes.	Maternal health	Citizens’ groups at the grassroots: village budget clubs at the parish level (12 women and eight men in each), district level councilors, and technocrats Targeting both the demand side and the supply side for maternal health care	Initiatives by CSOs dated back to 1999; the assessment took place in 2014, after completion of two-year cycles of interventions during 2010–12 in one area and 2012–14 in two areas.	Bamanyaki (2015) The first is a doctoral dissertation; the evaluation used an exploratory sequential mixed methods design: Qual and then Quant Qual Theory-based evaluation and process tracing; Case study Interviews with stakeholders, FGDs, community members, and reproductive age women, document review, observation. Quantitative study; quasi-experiment using a post-test treatment group and two sets of control groups; treatment group was participants residing in villages in the operational area of intervention; control – non-intervention villages; 500 respondents Bamanyaki & Holvoet (2016) is a journal article reporting only on the theory-based evaluation from the dissertation, providing the same information	Gender: Adoption of a district gender policy Health: Prioritisation of Women’s barriers to maternal health care in district plans; Gendered health: Increase in budget allocation for issues identified as gender barriers for maternal healthcare access; gender-responsive changes to service-delivery regulations; Quant: Health knowledge of maternal health services, rights and entitlements; utilisation of prenatal care; child delivery at a health facility; utilisation of skilled birth attendant; utilisation of postnatal care	Partially successful Women’s issues prioritised, and budgets increased at the district level (13.6% to 15.5% allocation for health from draft to approved budget); investment during 2013/14; equipment and infrastructure strengthening for maternal health. The successes could not be sustained; the commitment of district technocrats to gender-responsive policies, budgets, and service delivery decreased following FOWODE’s shift in focus to female councilors and grassroots citizens in 2010. The district gender policy, developed by technocrats in 2009, has never been presented to the district council for approval. Further, there was reduced zeal or a total lack of continuity in grassroots-level activities following the phase-out of direct support to grassroots women’s groups by the outside government GRB initiative. Technocrats The physical presence of a grassroots-level, outside-government GRB initiative in a sub-county significantly enhanced awareness among rural women, especially regarding maternal health rights and entitlements. Knowledge of maternal health services, rights, and entitlements was significantly associated with attendance of four or more prenatal care visits, but was not associated with utilisation of delivery care, delivery assisted by a skilled birth attendant, and postnatal care.
2	[18–21]	ONE MAN CAN, LIMPOPO, EASTERN CAPE, SOUTH AFRICA 4 publications: Dworkin et al., 2013, Van der Berg et al., 2013, Fleming et al., 2016, and Cazarin 2021						
		Objective: Reduce the spread and impact of HIV and AIDS, and reduce violence against women and men through changing gender norms about masculinity and promoting gender equality Interventions: a. The One Man Can (OMC) intervention: 6 workshops with groups of 15–20 men and intensive community mobilisation through Community Action Teams (CATs), religious leaders’ workshops, and campaigns for medium and long-term impact b. One Man Can (OMC) Fatherhood project: 5 workshops of 2 days’ duration each; intensive community mobilisation throughout the intervention period.	IPV, GBV, and HIV	Men, community, including religious leaders of both genders	2 years overall. Each cycle of workshops consisted of 5 sessions of 2-days’ each.	Dworkin et al. (2013) and Fleming et al. (2016) are qualitative evaluations of the OMC intervention in Limpopo and the Eastern Cape (2010), conducted through IDIs with 60 participants, no later than 6 months after they finished a group workshop. Each of these studies examined different outcomes. In-depth interviews with men no later than 6 months after they finished a group workshop. Cazarin 2021 assesses the outcomes of the OMC project’s engagement with religious leaders; Ethnography for 2 years, document review, observation 2017–18 Van der Berg 2013 et al., assess the OMC Fatherhood Project in the Eastern Cape, using qualitative IDIs with male participants once within six months post-intervention	(Qualitative self-reports) Gender: Changes in perception of women’s rights, seeking wives’ or girlfriends’ participation in household decision-making, participation in household tasks, and education in violence towards women, children, and other men. Changes in belief and practices about fatherhood from provider to carer, nurturer; improved communication with children Change in attitudes and willingness to engage in the prevention of SGBV by religious leaders Health: reduction in sexual partners, increased condom use, Willingness to be tested for HIV; Willingness to receive HIV-care and treatment if living with HIV Gendered Health: Reduction of harmful masculinities w.r.t. HIV-related behaviour: Increased capability to overcome masculinity-related barriers to testing/care/treatment. Ability to express vulnerability and discuss HIV openly with others	Partially successful as assessed through self-reports The evaluation showed a positive shift in men’s reported attitudes to **gender norms** and in practice in terms of awareness of women’s rights, participation in some household tasks, joint decision-making within the household, and more care than violence for women. Reported changes **in health behaviours** included a reduction in the number of sexual partners, increase in condom use. *At the same time, some men expressed ambivalence and concern that women would start controlling men* Reported increased capability to overcome masculinity-related barriers to testing/care/ treatment; increased ability to express vulnerability and discuss HIV openly with others, which led to greater willingness to be tested for HIV and receive HIV care and treatment for those who were living with HIV Religious leaders marginally successful Workshop participants developed new language and consciousness, engaged with tactical activism, but fell short on collective activism due to strategic silences about SGBV Fatherhood project partially successful Men described a transition from a disciplinarian and provider role towards one of increased involvement, companionship, nurturing, and affection towards children. There was also reduced alcohol use, safer sex, and reductions in male violence against both women and children. *Not all men were able to achieve these changes; peer pressure and the inability to play the role of an effective breadwinner were challenges*
3	[22–24]	**ONE MAN CAN, MPUMALANGA, SOUTH AFRICA. 3 publications: Pettifor et al., 2018, MacPhail et al., 2019, Treves-Kagan, et al., 2020.**						
		Objective: Changing gender norms about masculinity to prevent IPV and HIV risk behaviours. Intervention: One Man Can (OMC): 5 workshops of 2 days’ duration each; intensive community mobilisation throughout the intervention period. The intervention was delivered through local community mobilizers and through volunteer cadres called Community Action Teams (CATs), who were trained to support activities in each community. Intervention activities comprised workshops, community activities and leadership engagement open to men and women, with workshops and activity content focused on seven areas; (1) gender, power, and health, (2) gender and violence, (3) alcohol abuse, (4) gender, HIV and AIDS, (5) healthy relationships, (6) human rights, and (7) taking action for change. Mobilizers and CAT members created opportunities for community dialogue about these themes within and outside of workshops through community activities including door-to-door home visits, street soccer and soccer tournaments, mural design, and discussions, facilitated discussions in venues where men gather, including shabeens/ bars, and film screenings with a thematic discussion to follow, and community theatre. The team also engaged formal leadership and community organizations in dialogue on the intervention content and sought support for intervention activities.	IPV, HIV	Young adults (18–35 yrs.) with a focus on men for workshops Community members	2 years (2012–14); 5 sessions of 2 days for each group.	MacPhail et al., 2019 is a qualitative process-evaluation. It included longitudinal qualitative Interviews with community members (*n* = 52) at 6, 12, and 18 months into the intervention; mobilisers (*n* = 26) at 6 and 18 months; FGDs with volunteer CAT (*n* = 22) at 6 and 18 months to explore perceptions of the change process of individuals and communities. Treves-Kagan et al., 2020 is a longitudinal qualitative study carried out over a period of 18 months during the intervention: in-depth interviews at two time points with 15 community-mobilisers, at three time points with 19 community members; FGDs at two time points with 11 community-action teams (124 members) Pettifor et al., 2018 is a quantitative end-line community cluster-randomised trial	Gender Process through which the shift to Gender equitable attitudes occurs. Perceptions of personal and community-level change regarding gender norms and violence Change in gender attitudes using the Gender Equitable Men’s Scale (GEMS) Perpetration of IPV in the last 12 months Health Having two or more sexual partners in the last 12 months, condom use at last sex, and recent hazardous/harmful drinking assessed using the Alcohol Use Disorder Identification Test.	Marginally successful Qualitative reports from intervention staff and community members indicated that the shift towards gender-equitable attitudes happened when men developed greater respect and empathy for their partners, and communication between couples improved. Participants self-reported changes in their gender-equitable attitudes and use of violence because of participation in the programme. However, they also reported that sustaining non-violent behaviour was a struggle, and that there was resistance to equitable gender norms from many in the community. There were self-reported changes in attitudes towards gender norms among men, but not women, using the GEM scale. There were no changes in IPV, condom use, or hazardous drinking.
4	[25,26]	**TSIMA (ONE MAN CAN ADAPTED WITH AN HIV FOCUS) 2 publications: Gottert et al., 2020 and Leddy et al., 2021**						
		Objectives: Increase HIV testing, linkage to and retention in care and treatment among men and women ages 18–49, by addressing key social barriers to service uptake – including inequitable gender attitudes and norms. Intervention: Community mobilisation intervention; adapted from Sonke’s program *One Man Can*, with substantial changes made to (1) add more emphasis on HIV testing and treatment uptake (the main trial endpoints), and (2) equally engage and engender change among both men (the main target population for *One Man Can*) and women. Activities comprised five two-day workshops (with about 10–30 male and female participants), community-based activities (e.g. street theatre; murals; meetings with local leaders), young women’s groups, support groups for people living with HIV (PLHIV), and engaging village leadership and other stakeholders.	IPV, HIV	Women and men ages 18–49, Community members	3 years 2014–2018 5 sessions of 2 days’ each for each group	Leddy et al., 2021 is a qualitative assessment: 55 in-depth interviews with community members (48% women) and staff/community leaders (70% women), carried out during the last 3 months of intervention Gottert et al., 2020 is a mixed-methods evaluation. The quantitative study was carried out at baseline and end line; the qualitative study was carried out at end line, and a year later, after preliminary analysis of the quantitative end line survey. In-depth interviews (IDIs) with 23 intervention staff (community mobilisers and Community Action Team members) and 59 community members who had participated in the workshops; also, two focus group discussions (FGDs) with a total of 11 community mobilisers (10 of whom completed previous IDIs).	Gender: Endorsement of equitable gender norms assessed using GEM; IPV perpetration or experience Qualitative: shifts in gender norms about a) male toughness b) IPV c) control over women d) health being women’s sole responsibility and possible reasons for the shifts, including the intervention Health a) Information about the health and preventive benefits of early ART; info about sero-discordance and why both partners should be tested; Increased HIV uptake Gendered Health Couple support for the uptake of HIV services	**Positive changes, but null intervention effect** Promoting critical reflection around several specific gender norms, coupled with information (e.g. benefits of ART) and skill-building (e.g. communication), was perceived to support men’s and women’s engagement in HIV services. In both intervention and control communities, there were significant increases in self-reported endorsement of equitable gender norms – norms that were quite inequitable at baseline and reduction in perpetration of IPV. No intervention effect could be discerned. The most plausible reason that emerged for the broad shifts in gender norms (and the lack of evidence that these shifts were due to intervention activities) was rapidly increasing access to TV programming (via satellite dish) and smartphones Younger men in both intervention and control communities reported reductions in IPV perpetration, leading to a null intervention effect.
5	[27,28]	**UJANA SALAMA ADOLESCENT CASH PLUS INTERVENTION, TANZANIA. 2 publications**: **Chzhen et al., 2021 and Waidler et al., 2025**						
		Objective: to improve gender equitable attitudes among adolescent boys and girls. Intervention: *Ujana Salama* complemented the Government-implemented bimonthly Conditional Cash Transfer for school attendance and health check-ups. Face-to-face 2-hour weekly sessions for 12 weeks in mixed sex groups, providing livelihood and life skills training; followed by 9-month mentoring through meetings twice a month to coach on livelihood options. Supply-side strengthening of adolescent-friendly HIV and sexual and reproductive health (SRH) services. At the end of the mentoring, adolescents received 80 USD equivalent cash to pursue education, vocational training, or business start-up.	Adolescent SRH, HIV	Adolescent boys and girls (14–19 years)	2018–2019 18 months. Each cycle of workshops is of 24 hours.	Chzhen et al., 2021 is a cluster randomised trial, consisting of midline and end-line surveys with adolescents and providers Waidler et al., 2025 is a quantitative survey conducted 22 months after the intervention ended	Gender Gender attitudes using the Gender Equal Men (GEM) scale Relationship status: ever married, sexual debut, age at sexual debut, first sex forced; experience/ perpetration of violence Health: Whether experienced a pregnancy; Knowledge and use of modern contraception; use of reproductive health services, HIV testing, HIV knowledge, HIV risk perception; depressive symptoms, stress. Health service readiness using the Service Availability and Readiness Assessment (SARA) guidelines	Not successful Limited evidence that a government cash plus intervention targeted to adolescents improved gender equitable attitudes. The ‘cash plus’ life skills training did not have an impact on the full 24-item GEM scale after 12 months (end line), although it had an impact of .88 points (*p* < .05) soon after the training, at midline. The intervention did increase gender- equitable attitudes related to the domestic chores and daily life domain, but there were no significant impacts on the remaining three subscales at midline or end-line: i.e. support for equality in relationships, equal reproductive health/STI prevention, opposition to violence against women, and opposition to homophobia/violence against gay men Post-intervention impacts. Protective impacts were found on lifetime sexual violence risk among females and SRH health-seeking among males. In addition, we found two new impacts emerging that were not detected at previous rounds, including reduced self-perceived HIV risk, increases in marriage and pregnancy (of their partners) among males, and adverse effects on depressive symptoms (impacts on mental health estimated at Round 3 were protective). Finally, impacts on HIV testing, violence perpetration, gender equitable attitudes, contraceptive knowledge, and HIV prevention knowledge found at earlier rounds were not sustained.
6	[29–31]	**BANDEBEREHO INTERVENTIONS, RWANDA. 3 publications: Doyle et al., 2014, Doyle et al., 2018, Doyle et al., 2023.**						
		Objectives: To increase men’s participation in care work and in MNCH, SRHR, and violence prevention Intervention: Men Care+ intervention (Bandebereho), RWANDA Participatory, small group sessions of critical reflection and dialogue. 15 sessions for fathers’ groups; 6 of these attended by their spouses. Sessions take place once or twice weekly and are 3 hours each Community volunteers (local fathers) met with the same group of 12 men/couples on a weekly basis. Three intervention cycles, each with 570–576 couples.	MNCH, SRHR, IPV	Young adult men aged 18–35 who are either currently expecting a child or are already fathers to children aged under five, and their spouses	March 2014 - July 2015 Each cycle of workshops is of 45 to 48 hours.	Doyle et al., 2014 is a qualitative end-line assessment of the first intervention cycle, consisting of public testimonies by participants and facilitators. Doyle et al., 2018 is a baseline and 21 months post-intervention quantitative RCT assessment of the third cycle of intervention. Doyle et al., 2023 is a quantitative assessment of the third cycle of interventions, 6 years post-intervention.	Gender: Experienced physical /sexual violence from partner in past 12 months; Sharing of childcare and household tasks; Time spent on childcare and household tasks; Man has final say on household’s weekly/ monthly income and expenses: Man has final say on how many children to have or spacing of children Health: Mean number of ANC visits women attended; Mean number of ANC visits accompanied by man; Perceived partner support during pregnancy; % Used modern contraception	Successful as assessed by self-reports More men (than before the intervention) reported being involved in house chores like feeding, washing, and taking care of infants, a role entirely reserved for women. There was an increase in self-reports of accompanying their wives to deliver their babies and taking a sick child to the health center. Significant increase in open dialogue between couples, especially on financial matters. Quantitative survey showed 21 months post-intervention showed, in comparison to the control groups, substantial improvements in multiple reported outcomes, including women’s experience of physical and sexual IPV, women’s ANC attendance, men’s accompaniment at ANC, modern contraceptive use, and partner support during pregnancy. Importantly, the intervention also led to reductions in men’s dominance in household decision-making and improvements in the household division of labor. All positive outcomes were sustained 6 years post-intervention, showing statistically significant differences between intervention and comparison groups in reported physical, sexual, economic, and emotional IPV; Intervention couples report fewer depressive symptoms, less harmful alcohol use, and improved maternal health-seeking, father engagement, and division of household labor and decision-making.
7	[32–34]	**INDASHYIKIRWA INTERVENTION, RWANDA. 3 publications: Stern et al., 2019**, **McLean et al., 2020, Dunkle et al., 2020**						
		Objectives: build skills for healthy, equitable relationships and to shift the beliefs, behaviours, and norms that underpin male dominance and violence in relationships. Intervention*: Indashyikirwa* couples training workshops for 840 heterosexual couples. The core curriculum covered concepts of power and gender; human rights; managing triggers of IPV – including alcohol abuse, jealousy, and economic stress; healthy relationships; activism and providing empowering responses to those experiencing IPV. Shorter duration preparatory workshops for community leaders to enable their active involvement. Community outreach by trained community activists. Provision of support to victims through the creation of women’s safe spaces.’	Sexual coercion, IPV	Couples, community	4-year intervention: 2015–2018. Each cycle of workshops was consisted of 21 sessions implemented over 5 months	Stern et al., 2019 and McLean et al., 2020 are based on a longitudinal qualitative process evaluation of 14 couples, in Nov 2015, before starting, immediately after completing sessions in May 2016, and one year later in 2017. Stern et al., 2019 focused on changes in attitudes to consensual sex and sexual coercion; McLean 2020 examined changes in gender norms and roles in couples’ relationships. Dunkle et al., 2020 is based on a quantitative Community RCT 17 months after completion of all workshop sessions. In all three, both men and women interviewed.	Gender: Experiences of coerced sex; sexual communication between partners; acceptability for women to initiate sex. Shifts in relationship dynamics: men’s engagement in bringing in more money and domestic duties; women’s participation in household decision making, and women’s access to economic resources. Experience of physical/sexual/emotional violence or economic abuse in the past 12 months by current male partner; Help-seeking among IPV survivors in the past 12 months; Quality of relationship with main partner. Health: a) Depressive symptoms (men) b) Problematic alcohol-use (men) c) self-rated health (women) d) PTSD symptoms (women) e) Problematic alcohol-use (women)	**Partially successful** Quantitative data showed substantial and statistically significant reductions in experiences of physical and/or sexual IPV shown after 24 months of follow-up among both men and women, and in all health outcomes Qualitative data showed mixed results. There were moderate but positive shifts in all aspects of the relationship dynamics. However, the data also show that these ‘shifts’ occurred without fully transforming deeply entrenched beliefs and norms around gender roles and male authority over economic resources. Post-intervention, men still believed that these tasks are fundamentally the woman’s responsibility, but that a man could choose to help on his own terms. Women also seemed to agree that domestic duties were still ultimately the primary responsibility of women. While there was increased transparency and discussion around decisions over income and assets, men retained control of the decision-making process and had the final say.
8	[35]	**MAP INTERVENTION, KABALE, UGANDA. 1 publication: Ghanotakis et al., 2017**						
		Objectives: To foster gender equitable attitudes; increase use of dual methods of contraception among HIV care& treatment male clients. Intervention: 10 sessions or workshops for men using a curriculum adapted from MAP – Men as Partners interventions delivered by peer educators who received a 2-week-long training. Training for providers for improved counselling, and included gender training	HIV and contraception	Men	The duration over which the 10 sessions were delivered is not specified	Quantitative pre-post sub-analysis within a larger RCT of the male peer educators and group members. 6 months after all workshops were completed.	Gender Changes in gender attitudes assessed using the GEM scale. Health: Increased use of dual methods of family planning among HIV care & treatment clients.	Not successful No significant change in the overall GEM scale post-intervention. Scores for a few selected GEM Scale items, like attitudes to violence against women, improved. Respondents in the pre- and post-intervention survey rounds expressed simultaneous support for both gender inequitable and gender equitable norms.
9	[36–38]	**STEPPINGSTONES & CREATING FUTURES, SOUTH AFRICA. 3 publications: Jewekes et al., 2014, Gibbs et al., 2015, Gibbs et al., 2018**						
		Objectives: Forge positive masculinities – reduction in IPV, greater participation in household and care work, joint decision-making, and improving men’s economic livelihoods. Intervention: *SteppingStones* is a behavioural intervention combining HIV-prevention with the pursuit of greater gender equality. Sessions involve single-sex groups. Topics include communication, assertiveness, reducing gender violence, sex, and love. *Creating Futures* helped participants find work or set up a business. Topics include securing and keeping jobs, writing CVs, and budgeting and saving. Implemented in two urban informal settlements of **Kwa-Zulu-Natal** with 232 out-of-school youth: 110 men and 122 women	IPV and HIV	Young men and women	2012–2014 21 sessions of 3 hours each	Jewekes et al., 2014, analyse results from the quantitative baseline surveys and surveys at 28- and 58-week post baseline of a pilot intervention Gibbs et al., 2015 report on a qualitative longitudinal cohort study during the pilot intervention: at three time points with men, and two time points with the main female partners. Gibbs et al., 2018 report on qualitative in-depth interviews post-intervention: 11 men at 3- and 9-months post interventions; with the main sexual partners of 11 men at 9 months post intervention; 3 focus groups with 34 men at the end of the final session.	Gender: Quantitative: Experience/perpetration of physical and/or sexual violence during the past 3 months; Change in gender attitudes using the GEM; men’s controlling practices Qualitative Changes in attitudes to gender equality and roles; Reduction in perpetration of violence; improved communication with the primary partner; greater participation in childcare and related domestic activities Health: Prevalence of depression symptoms; prevalence of suicidal ideation Reduction in alcohol use. Economic: Earnings in the past month	Mixed results in the quantitative assessment, and marginally successful according to the qualitative assessments Significant reduction in women’s reported experience of the combined measure of physical and/or sexual IPV in the prior three months. But men did not report a reduction in violence perpetration, and there was no reduction in non-partner rape. Both men and women scored significantly better on GEM. The prevalence of moderate or severe depression symptomatology among men and suicidal thoughts decreased significantly. There was an improvement in the men’s economic situation However, case studies found there was no change but only a slight shift away from harmful youth masculinity. e.g. there were shifts in gender norms and relationships towards improved communication and avoiding conflict. At the same time, some men continued to seek multiple-sexual partners and continued using violence against partners. They were ridiculed by their peers for attempting to change their masculine behaviours.
10	[39–41]	**MAISHA INTERVENTION, TANZANIA. 3 publications: Kapiga et al., 2019**, **Harvey et al., 2021, Lees et al., 2021**						
		Objective: Reduction of IPV experienced and changing women’s acceptance and tolerance of IPV; attitudes and beliefs Intervention: 10 sessions delivered to women not formally employed and not previously part of any microfinance group. Known as the MAISHA intervention, following the Wanawake na Maisha (‘women and life’ in Swahili) curriculum developed by Engender Health	IPV	Women participating in a group micro-financing scheme	September 2014 – January 2018; each group attended 10 sessions over 20 weeks.	Lees et al., 2021 is a qualitative longitudinal study: IDIs with 18 participants, 12 from the intervention arm and 6 from the control arm; 12 FGDs (9 in intervention +3 in control arms). At three time-points: Baseline, within one-month post-intervention, and 2 years post-intervention Kapiga et al., 2019, and Harvey et al., 2021 are Clustered RCTs carried out 24 and 29 months after the intervention	Gender a) Experience of physical/sexual/ emotional IPV by current or any other partner in the past 12 months b) Attitudes accepting of IPV c) believes IPV is a private matter d) Believes a woman should tolerate violence to keep the family together e) Disclosed intimate partner violence in past year (among those experiencing physical or sexual intimate partner violence in past year) Qualitative: Increase in women’s understanding of gender as a social construct and of male power; gaining confidence; improving communication through conflict management and boundary-setting; engendering change for children and other women	Marginally successful Increase in women’s understanding and awareness of the non-acceptability of IPV; they were less tolerant of IPV, and more confident about challenging violence. However, there was no significant impact on reducing or preventing physical or sexual IPV.
11	[42–44]	**MEN CARE + INTERVENTION, SOUTH AFRICA. 3 publications: Foundation for Professional Development (FPD) 2016, de Wit 2016, Kedde et al., 2018**						
		Objective: a) To foster gender equitable attitudes among young men, positive attitudes towards contraceptive use, and increase condom use. b) To engage men, between the ages of 15 and 35 years, as caregiving partners in issues concerning Sexual, Reproductive, and Maternal Health and Rights. Intervention: a) Group Education with young men in 9 sessions covering SRH, substance use, HIV and IPV b) Group Education with fathers and partners: 11 sessions including child delivery, fatherhood, caregiving, sharing of care work c) Counselling and therapy work with men who have used violence; d) Workshops with health sector workers on the importance of engaging men in SRHR services; e) Community campaigns promoting MenCare+ to increase awareness of men’s roles in fatherhood and caregiving; f) Advocacy and alliance building with organisations and government on issues to do with engaging men.	SRMH	Young men (15–35 yrs.)	January 2013- December 2015 9–11 two-to-three-hour sessions for each group	FPD 2016 is the report of an outcome evaluation using mixed methods. The quantitative data were from pre- and post-questionnaires and included 270 men from the SRH group and 444 from the fathers’ group. The qualitative assessment consisted of FGDs with 54 participants and telephonic interviews with 35 programme stakeholders: staff, healthcare, and social workers. De Wit 2016 is a student thesis using mixed methods. The quantitative study included 104 men assessed at baseline and end line. The qualitative study consisted of IDIs with 6 fathers or fathers-to-be. Kedde et al., 2018 is a mixed-method evaluation of the young men participants in the SRH sessions. Quantitative study included a pre- and post-questionnaire survey with 265 men, and qualitative study included FGDs with 33 men.	Gender: Change in gender equitable attitudes using the GEM scale for young men Definition of the role of a young man; Perception of the father role (with factors from the Inventory of Father Involvement or IFI) Health: Use of condoms during last sex; accompanying partners for prenatal visits; couple communication about contraceptive methods; Attitudes towards contraceptive use, using the ‘Contraceptive Attitude Scale’. Use of SRH services Gendered Health: Shared decision-making with a partner on condom use.	Reported attitudinal change but no behavioural change. Improvement in GEM scores of young men and fathers; reported increase in respect for women partners by young men; fathers reported a change to a more egalitarian view of parental roles and equal power. Reported increase in condom use at last sex and increase in contraceptive use No significant behavioural change in terms of increase in caregiving roles; in ANC accompaniment; in presence during childbirth; health-facility related barriers cited
12	[45,46]	**SIBLING SUPPORT FOR ADOLESCENT GIRLS IN EMERGENCIES (SSAGE) INTERVENTION, NIGERIA. 2 publications: Koris et al., 2022 and Seff et al., 2022**						
		Objectives: To bring about more egalitarian and less violent family relationships and greater gender equality in a humanitarian setting Intervention: Delivered to four groups: adolescent girls & boys, and female and male caregivers. 12-session workshops for the adolescents and 13-session workshops for the caregivers.	GBV, IPV, sibling violence	Adolescent girls & boys; female and male caregivers in the family	4 months September – December 2020 Each cycle of 12–13 session-workshops lasting 36–39 hours	Both studies report post-intervention qualitative assessment using IDIs and FGDs with caregivers, paired interviews, and participatory activities using vignettes with adolescent boys and girls, and key informant interviews with programme staff	Gender a) Improved quality of communication between caregivers, between adolescent siblings, and between adolescents and caregivers within the family b) changes in perpetration of violence across family relationships c) shifts towards more egalitarian and less violent family relationships d) changes in attitudes and beliefs about the rights and protection of adolescent girls	Partially successful Participants reported shifts toward more egalitarian familial relationships, increased endorsement of girls’ rights, decreased violence perpetration, and improved communication within the families. However, aspects of normative, patriarchal norms governing the treatment of adolescent girls were maintained by participants.
13	[47,48]	**UNITE FOR A BETTER LIFE, ETHIOPIA. 2 publications: Sharma et al., et al., 2020 and Leight et al., 2021**						
		Objective: Reduction in women’s experience of/ men’s perpetration of past-year physical or sexual violence. Intervention: Unite for a Better Life (UBL) intervention delivered to single sex groups and to couples. 14 workshop sessions delivered twice a week	IPV, HIV	Men, women, and couples	7 weeks. April – October 2015 Workshop sessions lasting about 2–3 hours each	Sharma et al., 2020 report on a cluster RCT with four arms: men-only intervention, women-only intervention, couple intervention, and control. 24 months post-intervention Leight et al., 2021 report on a cluster RCT assessing the spill-over effect on non-participants in the intervention areas, 24 months post-intervention	Gender Women’s experience/male perpetration of past-year physical, sexual, or emotional IPV Health a) HIV awareness b) Condom use at last intercourse	Partially successful Effective in reducing self-reported perpetration of sexual IPV when delivered to men but did not reduce IPV when delivered to couples or women. There was evidence of increased HIV knowledge and condom use at last intercourse among women Indirect beneficiaries showed similar results as direct beneficiaries
14	[49–51]	**HOUSE VISITS TO PROMOTE MCH, BAUCHI, NIGERIA. 3 publications: Belaid et al.,2021, Mudi et al., 2021, Belaid et al., 2024**						
		Objectives: Improving men’s engagement with MCH; support for non-violent relationships with their wives. Intervention: Home visits by male and female community workers once in two months during pregnancy and immediately postpartum, and a final visit one year after the delivery.	MCH, IPV	Couples	5 years (2015–2020)	Belaid et al., 2021 report on a qualitative midterm evaluation using stories-of-change collected from 23 women and 21 men in households who had received home visits, from eight male and eight female home visitors, and four government officers attached to the home visits program. Mudi et al., 2021 report on an end-line qualitative assessment using the same ‘stories-of-change’ method Belaid et al., 2024 is a qualitative, immediate post-intervention evaluation assessing changes in attitudes to gender equity. Methods included nine key informant interviews with policymakers and 14 gender and age-stratified focus group discussions with men and women from visited households, with women and men home visitors and supervisors, and with men and women	Gender Increase in spousal communication, Changes in perception about intimate partner violence, and support for non-violent inter-spousal relationships Male support for household chores Women don’t need spouses’ permission to seek their own healthcare Health: Increases in knowledge about maternal and child health Gendered Health: A Greater sense of agency and confidence among women during delivery and pregnancy Men’s support for antenatal care, immunisation, and seeking help for danger signs in pregnancy/delivery/postpartum Men paying for healthcare and providing nutritious food	Partially successful While the midterm assessment indicated improvement in gender and health indicators, the end-line stories of change did not mention that improved attitudes translated into shared decision-making or increased autonomy for women. Many of the men’s stories described a continuing paternalistic, male-dominant position in decision-making. However, the 2024 study, also conducted at the end line, reported that all respondents considered the intervention to have positively impacted gender equity: increased spousal communication, greater male support for household chores, women’s autonomy to seek health care for themselves, and reported reduction in IPV.
15	[52]	**DIFFERENTIATED SERVICE DELIVERY MODELS (DSDM) FOR HIV, SOUTH AFRICA. 1 publication: Mukumbang 2021**						
		Objective: Refashioning hegemonic masculinities; improving men’s engagement with HIV services. Intervention: Three different service delivery models that used the Differentiated Service Delivery Model (DSDM) approaches to improve men’s participation in HIV service: facility-based adherence clubs (FACs), community-based adherence clubs (CACs), and quick pharmacy pick-ups (QPUPs).	HIV	Men	November 2015- June 2017	Qualitative 6 FGDs and 20 in-depth interviews with male participants, during the intervention period	Health Improved uptake of HIV testing, treatment, and care services Gendered Health Hegemonic masculinities refashioned to ART-friendly masculinities	Partially successful At the individual level, DSDMs helped men to adhere to their medication through a) a sense of cohesion: social support and feeling of connectedness b) sense of assurance because ART services were reliable, convenient, and stigma-free c) perceived usefulness because services were flexible, and delivery was quick But at the community level, DSDMs showed minimal significance to gender transformation in terms of changing local beliefs and practices relating to health-seeking behaviours. Particularly, regarding the refashioning of reputational masculinity (the marks of honor and status among fellow men) and respectability masculinity (being strong, resilient, and disease-free), DSDMs showed an insignificant role
16	[53]	**CERVICAL CANCER SCREENING – COUPLE ENGAGEMENT, NIGERIA. 1 publication: Okedo-Alex et al., 2021**						
		Objective: Improving uptake of cervical cancer screening through improving spousal communication. Intervention: Three-pronged: advocacy with community leaders and leaders of the men’s association; awareness creation through health education sessions of 1.5 hours with husbands; and monthly meeting-based reminders to the husbands to encourage their wives to undergo cervical cancer screening.	Cervical cancer	Husbands of women who were 18 years or older and belonged to a men’s association	The duration of the intervention is not mentioned	Mixed method before and after study; quantitative study using pre- and post-questionnaires with 245 men assessing dimensions of self-reported involvement in their spouses’ cervical cancer screening; data on cervical cancer collected from the screening registers. The qualitative study consisted of 5 key informant interviews, three months post-intervention	Health Cervical cancer screening uptake Gendered Health Cervical cancer screening discussion with spouses; providing emotional support to spouses for cervical cancer screening; accompanying spouses for cervical cancer screening; providing funding for spouses’ cervical cancer screening	Not successful Men’s interspousal discussion on cervical screening increased significantly according to self-reports. The main objective of increasing the uptake of cervical cancer screening was not achieved.
17	[54,55]	**GIRL EMPOWERMENT INTERVENTION, LIBERIA. 2 publications: Hallman et al., 2018 and Ozler et al., 2019**						
		Objective: Reduce sexual abuse among early adolescent girls (13–14 yrs.). Intervention: A mentorship programme consisting of a) weekly Life Skills training by trained local mentors, during which the girls learn about life skills and financial literacy for 32 weeks b) Monthly discussion groups for participants’ caregivers c) Training for local health and psychosocial care providers on how to improve and expand services for survivors of gender- based violence. d) Small deposits are made into the participants’ savings accounts to promote financial literacy. GE+ In one arm, a cash incentive is given to caregivers for girls’ participation	Sexual violence	Early adolescent girls (13–14 yrs.)	2 years (2015–17) Cycles of 39 weeks: 32 weekly sessions, plus 7 weeks of interactions with a mentor to prepare a community action event aimed at engaging community members	Hallman et al., 2018 is an unpublished evaluation report, and Ozler et al., 2019 is a journal article. Both report on a Cluster RCT with three arms: control, the Girl Empowerment Intervention, and the intervention plus an incentive for the girls’ participation provided to their caregivers. Baseline in 2015 and End-line in 2017–18, 9 months post-intervention.	Gender: Gender Equity Index assessing attitudes towards gender norms; Attitudes to IPV; Experience of sexual violence: non-consensual touching; pressure to have sex; attempted rape; rape Health: Life-skills index: HIV knowledge; health knowledge; knowledge of condom effectiveness; knowledge of healthy intimate relationships. SRH: never married; never had sex; never pregnant; number of partners during the last 12 months; and condom use at last sex. Psychological well-being: *Rosenberg Self-esteem scale*; the child version of the *Short Mood and Feelings* questionnaire; the *Children’s Revised Impact of Event (CRIES) Scale* to assess PTSD	Not successful The effect of the intervention in comparison to the control was small. Scores in the gender and life skills indices showed significant improvement. However, the programme objective was not achieved. Ever experienced sexual violence rose from 37% at baseline to 85% at end line. Only 53% sought help to cope with the experience, and of these, two-thirds went to family members. At the end of the line, 55% met or exceeded the threshold for PTSD.
18	[56]	**CAPACITY-BUILDING AND EMPOWERMENT INTERVENTION WITH NURSES ON COUNSELLING IPV SURVIVORS, SOUTH AFRICA. 1 publication: Sprague et al., 2020**						
		Objective: Improving Nurses’ skill in empowerment counselling for women experiencing IPV Intervention: Training, ongoing supervision, mentorship and feedback to nurses	IPV	Women nurses	2011–2016 30 hours plus ongoing mentoring for each participant	Qualitative study within an RCT. In-depth interviews and participant observation. 12–15 months post-intervention	Gender (a) Health provider perceptions of changes in their own agency related to patient care including changes in attitudes, behaviours, and knowledge (b) Health provider perception of enhanced agency of patients after taking part in nurse-led empowerment counselling	Successful as assessed through self-reports Intervention nurses reported their own self-efficacy in counseling pregnant women on IPV improved. Their relational and collective agency expanded.
19	[57]	**LEARNING CENTRE INITIATIVE, REPRODUCTIVE HEALTH UGANDA (LCIRHU). 1 publication: Stern et al., 2015**						
		Objectives: To promote men’s roles as clients: increasing access to relevant SRH services; role as equal partners: influencing attitudes towards gender equality in their relationships, and reducing perpetration of IPV; and their role as advocates for SRH: through peer educators encouraging men’s participation in the promotion and delivery of SRH services Intervention: The Learning Centre Initiative – Reproductive Health Uganda (LCIRHU) programme; Use of multiple communication strategies; community outreach; male targeted clinic days; sexual health couples’ counselling	SRH	Heterosexual men (18–54 years)	2012–2014: 2 years	Baseline and end-line evaluation. Baseline assessment through a quantitative survey with a random sample of 164 men eligible for participation in the intervention; End-line assessment through a qualitative study consisting of FGDs with male beneficiaries, their female partners, and peer educators; and KIIs with project staff and male beneficiaries.	Gender Improved gender equitable attitudes and roles Health Improved knowledge, greater demand for and access to men’s sexual and reproductive health services; beneficiaries advocating for increased male use of SRH services	Mostly successful according to self-reports Beneficiaries reported improved use of SRH services; sexual-health couples counselling and testing was reported to promote gender equality and open sexual decision making. The findings had insignificant results to conclude on how the intervention influenced the role of men as advocates.
20	[58,59]	**INVOLVING FATHERS AND GRANDMOTHERS IN INFANT FEEDING, KENYA. 2 publications: Mukuria et al., 2016 and Thuita et al., 2021**						
		Objective: To effect a change in the roles of men in infant and young childcare and feeding; to promote the provision of support to mothers by grandmothers to improve infant feeding practices Intervention: Bi-monthly workshops for fathers’ and grandmothers’ groups respectively in two different communities, in which participants received information on health and nutrition and were encouraged to provide social support to mothers	Infant feeding	Fathers with children 6–9 months of age and grandmothers	2012 6 months	Thuita et al., 2021 is a process evaluation study conducted during the last two months of the intervention, consisting of 18 FGDs − 8 with fathers’ dialogue groups and 10 with grandmothers’ dialogue groups. Mukuria et al., 2021 reports on a quasi-experimental quantitative evaluation with three groups: fathers’ groups, grandmothers’ groups, and a comparison group. Interviews were conducted with mothers, fathers and grandmothers during baseline and the same households were surveyed in the end-line.	Gender: Support to the mother, assessed using a *Social Support Index* with two dimensions: receipt of support by mothers and provision of support by grandmothers and fathers Health: Consistency of foods regularly consumed; Adherence to minimum acceptable diet in the past 24 hours; consumption of animal-source foods in the past 7 days. Gendered Health: Improved child feeding through father’s involvement in childcare and feeding Specific physical support action or material support action to mothers by fathers and grandmothers regarding infant feeding	Partially successful according to self-reports Fathers’ and grandmothers’ provision of material and physical support to mothers increased significantly in intervention areas. There was also a significant improvement in the consistency of foods regularly consumed by infants and in reported feeding of animal-source food during the past seven days. However, there was no significant increase in the reported adherence to minimum acceptable diet for infants during the past 24 hours between intervention and comparison areas.

In total 13 of the 20 gender-transformative interventions addressed gender-based violence (GBV) or IPV, eight addressed HIV, five focused on maternal and child health, five were sexual and reproductive health interventions with adolescents and young people, and one was an intervention to promote uptake of cervical cancer screening. Eight interventions targeted men, eight targeted men and women (couples exclusively in two instances), and only three were women or girls-only interventions. Barring three, the interventions consisted predominantly of training workshops (sometimes complemented by community mobilisation activities). Two-thirds of the interventions for which duration was specified (12 of 18) lasted 2 years or less, and interventions exceeding 2 years consisted of multiple workshops cycles, with no more than 6 months of intervention per participant group.

### What to assess

We have categorised the outcomes assessed to monitor the progress of gender-transformative health interventions into three categories: gender outcomes, health outcomes, and gendered health outcomes.

Gender outcomes encompass knowledge and attitudes regarding gender equality, as well as changes in gender-related behaviour. Gender-unequal attitudes and behaviour, such as support for wife-beating or experience/perpetration of intimate partner violence, are adverse gender outcomes, the absence or reduction in which signifies progress towards greater gender equality.

Health outcomes encompass knowledge, attitudes, and health-related behaviours, including healthcare utilisation and health status (e.g. the presence of signs and symptoms of specific health problems or risk factors for such problems).

We arrived at the category of gendered health outcomes when we identified outcomes that did not fit into either of the above categories but depended on positive changes in gender-equal attitudes and behaviours. Some examples of ‘gendered’ health outcomes include joint condom-use decision-making by couples, women’s disclosure of their HIV status to their male partners, or men’s support for antenatal care for their spouses. In other words, when a health outcome results from changes in a gender outcome, such as agency, autonomy, or power relations, we call it a ‘gendered’ health outcome.

When discussing what to assess when evaluating progress in gender-transformative SRH interventions, interview participants addressed a range of issues, including the types of outcomes measured, their temporal nature, and their context-specificity. These are summarised below and then compared with findings from an examination of the 20 intervention studies (See Table 2 for outcomes assessed or indicators used)

**Table 2.** Gender outcomes to be assessed/assessed in gender transformative health interventions – interview participants versus intervention studies.

Interview participants	Intervention studies
**MEN/BOYS**	
***Changes in attitudes (self-reported)*** NIL	***Changes in attitudes (self-reported)*** Attitudes to intimate partner violence and sharing of domestic chores and daily life, measured using the GEM scale Attitudes to gender equality Encouraging other men to adopt gender equitable attitudes Attitudes towards spousal control and intimate partner violence; support for non-violence in interspousal relationship Attitudes related women’s rights
***Changes in behaviours and perceptions*** Towards intimate partner (reported by partner/wife) Use of violence Support in household chores Contributing own income for wife’s enterprise Own behaviour (self-reported) Heavy alcohol use Engaging in transactional sex Non-use of condom Having multiple partners Seeking support for accessing HIV-testing	***Changes in behaviours (self-reported)*** Towards their intimate partner Perpetration of violence (sexual, physical; emotional); controlling behaviour Participation in household tasks Communication with wife/ primary partner Seeking partner-participation in decision-making; shift to joint decision-making Towards their children Increase in involvement in caring for children Shift from being an ‘absent’ father to one more ‘present’ Improved communication with children Using less corporal punishment for discipling Positively socialising to be respectful, gender-equitable, and to share household responsibilities with parents and sibling Perceptions towards fatherhood – shift from viewing it as a ‘provider’ role to being more caring and protective and less violent Perceived masculine-role stress (for young men and adolescent boys)
**WOMEN/GIRLS**	
***Changes in attitudes (Self-reported)*** (Women and girls living with disability) Acceptance of self as a person Having self-confidence	***Changes in attitudes (Self-reported)*** Improvement in attitudes towards gender equality Attitudes less accepting of IPV; less acceptance that a girl/woman should tolerate violence to keep her family together; that IPV is a private matter
***Changes in behaviour*** Reports intimate partner violence to the police Able to leave an abusive relationship Rejoins school after dropping out due to pregnancy Negotiates condom use with partner Women mobilise mobilise themselves to ensure that perpetrators of violence are brought to justice (Adolescent mothers) Back in school and remains there after dropping out due to pregnancy Does well in school Interacts well with peers Participates in public spaces (Women and girls living with disabilities) Participates in community events Contesta for leadership positions in local institutions Receives community support for leadership	
***Changes in access to resources*** Starting an enterprise; generating income and using the income for reinvestment and expansion (objectively verified)	***Changes in access to resources*** (Objectively verified) Enhanced life skills among the girls (Assessed using tools) Empowerment

#### The need to assess/measure health-related as well as gender-related outcomes

Some interview participants emphasised the importance of evaluating progress in both gender outcomes and health outcomes in gender-transformative interventions (P22, P27). One of them had learned this from his experience with the Husbands’ School intervention. Initially, this intervention assessed only health outcomes, including changes in prenatal and postnatal consultations, assisted deliveries, and family planning use. The project team then realised the need to determine if these health outcomes were achieved alongside changes in gender-based inequalities.

*And so they had what they called ‘Husbands’ Schools,’ in which men facilitated or encouraged their wives to go to clinics or hospitals. But (xx) the organisation’s objective at the time with the ‘Husbands’ Schools’ was much more to improve health indicators in terms of maternal health, contraceptive use, and so on. It worked quite well. … It’s been a success in terms of health indicators’ improvement, but not much in terms of gender indicators’ improvement. So gender equality, positive masculinity, strengthening the ‘Women’s Agency,’ i.e. women’s decision-making, and all that (*P22).

The Husbands’ School project then included the following gender outcomes for monitoring: whether the woman is in a favourable family environment, whether her rights are respected, and whether their children’s rights are respected (P22).

Gender outcomes at the individual level, as assessed by the interview participants, primarily concerned women’s agency, autonomy, and decision-making power. Few participants mentioned changes in male attitudes and behaviour towards greater gender equality as gender outcomes to be assessed. Health outcomes that interview participants had used comprised mainly of the utilisation of maternal health and contraceptive services, the experience of gender-based violence (GBV), and access to GBV services. Additionally, a participant working with girls and women with disabilities mentioned their overall wellbeing.

Turning now to the 20 intervention studies, many assessed more than one outcome category (gender, health, or ‘gendered’ health). The vast majority − 18 out of 20 assessed gender outcomes, 17 assessed health outcomes, and 8 assessed gendered health outcomes (though they did not label them as such).

It is interesting to note that, in contrast to findings from interview participants, only four of the 20 intervention studies considered positive changes in women’s attitudes toward gender equality or in their empowerment and agency in assessing gender outcomes. They assessed positive changes towards gender-equal attitudes in men and changes in gender-unequal behaviour, including the perpetration of intimate partner violence by men or a lower incidence of intimate partner violence or sexual violence in women and girls.

Of the 18 studies assessing gender outcomes, three did not consider changes in health outcomes. It is encouraging that all the studies assessing health outcomes also assessed gender outcomes, in line with the understanding that both gender and health outcomes should be considered when assessing gender-transformative health interventions.

**Figure 1.**
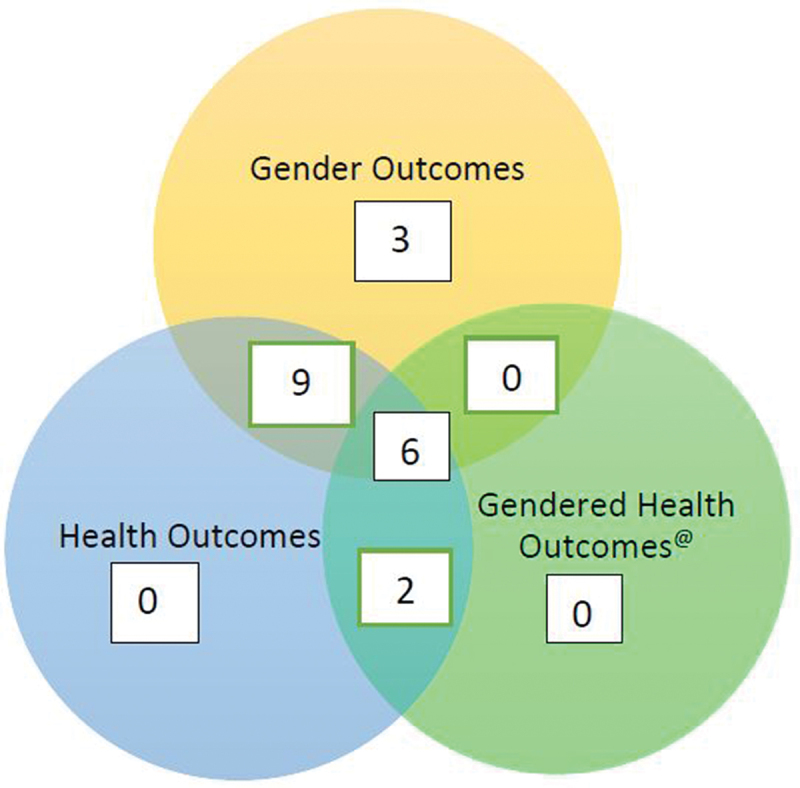
Distribution of the 20 evaluated interventions by the categories of gender-transformative health outcomes assessed (*N* = 20).

The distribution of the 20 evaluated interventions by the categories of outcomes they assessed is given in Figure 1. The figure also depicts the multiple outcome categories assessed in each study.

@Health outcomes that result from positive changes in gender-equal attitudes and/or behaviour.

#### Outcomes covering various facets, levels, and domains

Responses from interview participants also reflected the complexity of selecting appropriate outcomes to assess gender-transformative interventions.

For example, some participants stated that assessing not only outcomes but also processes was essential to determine what changes occurred and how they occurred:

… evaluating the entire process, … not only an initial evaluation, but also during the program, to understand how things are changing, and at the end of the program. So I think it’s important to have this approach. (P3)

However, only 9 of the 20 interventions in the review of published studies documented the change process (including 1 that assessed outcomes), while 11 evaluated only outcomes. Of these nine interventions, qualitative process evaluation studies [22,25,26,33] for three interventions offered insights on the mechanisms and pathways through which attitudes toward gender equality shifted. For the remaining six interventions, process documentation captured whether and among whom attitudinal and/or behavioural changes occurred at various stages of the interventions.

Speaking specifically about gender outcomes, participants listed indicators of behavioural change as the preferred outcomes they used/would use to assess progress in gender-transformative change (Table 2). Two participants expressed diverging views on the merits of assessing behavioural versus attitudinal changes related to gender equality. One participant believed that assessing gender-transformative change was best achieved by tracking behavioural change, as it was easier to measure than attitudinal change. According to her, behavioural change was also more tangible to observe and likely to be sustained. Her views were based on her experiences with male-engagement interventions to prevent intimate partner violence.

I find behaviour much easier to measure than attitudes, and it is often more impactful. For example, the use of violence, heavy alcohol consumption, having transactional sexual relationships, non-use of condoms, and having multiple partners. That sort of clustering of male behaviour that we know is associated with exploitative masculinities. And then, … … evaluation using a questionnaire that measures these different behaviours. If you see the whole group then changing, then what we are looking at is a gender-transformative intervention (P16)

However, another participant (P8) thought it was insufficient to assess behaviour change, as it could be transient. Attitudes and beliefs shape behaviour, and changes in these would be necessary measures of gender-transformative change (P8).

If we focus on the gender-related outcomes assessed in the evaluated interventions (*N* = 18/20), we find that 14 assessed either behavioural change alone (4) or both behavioural and attitudinal change (10), while 4 only assessed attitudinal change. However, in almost all studies assessing both gender-related behavioural and attitudinal changes, the reduction in the perpetration/experience of IPV was the behavioural change outcome of focus, rather than other manifestations of changes in gender norms or power equations within couples. Only four interventions examining both health and gender outcomes assessed attitudes alone. The reason is that health outcomes are frequently assessed based on behavioural change, so we often find changes in gender-equal attitudes assessed alongside changes in health-related behaviour. It is essential to note that, in most cases, the assessed attitudinal and behavioural changes were based on self-reports.

#### Assessing changes at multiple levels

Gender-transformative change encompasses changes at the individual, interpersonal, household, community, organisational, and structural (policy and institutional) levels. While many participants referred to individual and interpersonal gender-transformative changes, some working in regional organisations emphasised the need to recognise and measure gender-transformative changes at the organisational and structural levels:

We have defined two critical indicators at the health system level to assess gender-responsiveness, which have been accepted by the Ministry and included in the Health Information System … .One is the number of health facilities that have the readiness to provide GBV and sexual violence services at the outcome level, and the second is the number of users of GBV services disaggregated by location, age, sex, and disability, as an impact indicator. (P25)

Other examples of outcomes beyond the individual and interpersonal levels included the assessment of research projects (e.g. use of research evidence to tailor context-specific gender-transformative interventions), educational institutions (e.g. the existence of structures to address gender discrimination and provide redress), and community-based organisations (e.g. policies and interventions to promote participation by women and girls living with a disability) (P9, P26, P28).

In contrast to the observations by interview participants, most (13/20) evaluated interventions assessed changes solely at the individual level. Five assessed changes at the interpersonal level within the household (alongside individual changes), one assessed community-level changes, and one assessed policy changes increasing budgetary allocations towards gender equality. It is interesting to note that none of the interventions to prevent gender-based violence measured outcomes related to health sector changes, including the availability of services for gender-based violence. The only health sector-based study examined changes in the knowledge and skills of individual healthcare providers rather than institutional changes.

#### Choosing context-specific outcomes

Deciding on ‘What’ to measure or assess was challenging because conceptualising what constitutes gender-transformative change may not be the same across contexts, but rather manifest differently in different settings (P1). For example, entry into paid employment may indicate empowerment for some women but result in impoverishment and disempowerment for others, given the terms of employment.

Careful consideration of the assessment’s specifics and the selection of indicators tailored to the context was emphasised. Additionally, inequalities and differences within groups of women and men were also taken into account.

Sometimes people think that the fact that we have achieved gender parity in the number of participants shows that we have been gender-sensitive, or at least that we are on the path of transformation, but no if you targeted women who were not really in need in the community, not the ones whose skill change was going to make an impact at the community level if you targeted the wrong men as well, you didn’t achieve your goal. So, these indicators have to be well thought out, not just regarding men and women, but also which men and which women. (P1)

The intervention studies give no evidence of developing context-specific indicators or of considering intersectional inequalities. Most studies used standard indicators of gender attitudes and behaviour, as well as of attitudes and experience/perpetration of IPV, and none of the evaluations disaggregated the results by age, ethnicity, class, and so on.

### How to assess

#### The process of decision-making on what, how, and when to assess

Interview participants had varying perspectives on who decides on the process for determining what to measure. Significant differences in underlying epistemological worldviews often guided these.

A few recommended a priori theory-based approaches, in which decisions are made by the research or implementation teams. One would start by developing the theoretical basis for the drivers of change in a particular set of attitudes or behaviours, then develop tools to measure these attitudes and behaviours, test them, and finalise them for use (P8). This approach would call for the development of measurement or assessment tools during intervention design (P3).

Others believed that indicators and tools for measurement ought to be developed in consultation with the community where the intervention is to take place:

Talk to the people for whom the program is being designed. Remember, it’s always about people’s lived experiences. This is the only way one can understand the multiple domains in which change has to happen. (P19)

Constituting a committee of stakeholders was recommended by another participant as a way of tracking and fine-tuning an intervention throughout its implementation:

These are not the people implementing, but you want to check back with them, call them into a meeting, and tell them, This is how far we are going, and this is where we want to go. What do you think about it’? And then they bring their input to bear. And in that case, you have a better chance of achieving the kind of change you want to see because you are taking a different perspective on board. (P8)

Whether decided by researchers or with community participation, one participant emphasised the need to be flexible in choosing indicators, allowing new indicators to emerge during the intervention rather than relying on an a priori list (P12).

However, the ground reality was that implementers were often not free to decide what to measure and how to identify the indicators. Donors usually choose the indicators for monitoring. Some had a global indicators framework to which project managers were required to report (P18). In other instances, donor requirements led to multiple, siloed assessments, preventing a comprehensive understanding of the gender-transformative changes in the community as a whole:

We have different tools and different frameworks that are project-based. These depend on the project monitoring and evaluation framework agreed upon between the organisation and the specific donor. This is a challenge because we may not have a comprehensive assessment that provides the overall picture of the changes in the lives of girls and women with disability that we are working with. It is just project-based. If it is economic empowerment, then this project is assessing the baseline and endline of the economic component, using the set of indicators we have agreed with the donors (P28).

Few details are available on how the 20 evaluated interventions developed their assessment indicators. All the evaluations appear to have been conducted by the research team members, who differed from the implementor team. None of the 20 interventions mentioned the involvement of community members or intervention participants at any stage of the evaluation study’s planning or analysis, except as respondents.

#### Study designs and tools

The processes adopted guided the study design and tools used to assess gender-transformative changes in the implemented interventions. Some believed that randomised controlled trials (RCTs) were the gold standard for ‘objectively’ evaluating the impact of any intervention, including gender-transformative interventions (P22). The quantitative scales commonly used by participants to measure gender outcomes included the Gender-Equitable Men (GEM) scale and the Gender Role Attitude Scale (GRAS). Both these assess attitudinal changes alone.

One participant highlighted the value of a longitudinal qualitative method:

We have been keen on using longitudinal qualitative methods, … .repeatedly interview small groups of participants from before and all the way through the interventions and then afterwards to sort of try and get a sense of impact on their lives, as well as to get a sense of how much they find the intervention useful, and what bits might be more or less useful … . to have a look at what changes and how we see change …  (P16).

Many other participants reported using qualitative and participatory approaches, such as community consultations and interactions with intervention participants, to understand their perspectives on what had changed as a result of the intervention, whether the changes were positive or not, and why (P12, P28). Tools used by participants for qualitative assessments included, in addition to in-depth interviews and focus group discussions, a range of methods such as photo voices, narratives about the most important change that had come about in their lives and in their communities, and outcomes-harvesting based on people’s stories.

But also, we have personal stories, individual stories of what has been changing. This is another way to measure our achievements. There are also stories of community-level changes. When we run an awareness session with the community, we later go back to discuss what happened after the session. They provide feedback. We have another feedback framework. After the sessions, we received some calls from the community, ‘You know, after that training, when I went back, I saw a girl like this. How can I handle that case’? (P 28)

Some participants believed that participatory approaches involving community members in a continuous process would help identify markers of success that may not emerge during structured midline or endline assessments.

These communities also have their indicators of how they see change. And when it was this thing they said (positive sexual relations as an indicator of more equal gender relations), I still kept laughing, but we didn’t even think about it … When they kept talking about it, I said, ‘Hey, wait a minute.’ I can assure you that if you go with the real questionnaire at the endline and you ask these people what the best things are, whatever has happened, I think you may not hear anyone talking about sexual relationships. (P12)

Most others who responded chose a middle path and recommended combining quantitative experimental or quasi-experimental study designs with qualitative approaches, including interviews, focus group discussions, community consultations, and community-based observations involving multiple stakeholders (P3, P16, P18).

Of the 20 interventions evaluated across the 44 published studies analysed, 11 used both qualitative and quantitative evaluation methods. In six of these, stand-alone quantitative experimental or quasi-experimental evaluations were complemented by separate qualitative assessments, whereas five interventions used mixed-methods evaluation studies. Of the remaining nine interventions, five were evaluated exclusively using qualitative methods, and four were evaluated quantitatively (Table 3).

**Table 3.** Assessing gender-transformative health interventions: interview participants’ perspectives versus intervention studies’ practice.

Interview participants	Intervention studies
**WHAT TO ASSESS**	
Gender as well as health outcomes ‘Gendered’ health outcomes not mentioned Women’s empowerment as the focus in gender outcomes	18/20 assessed both gender and health outcomes. 8/20 assessed ‘gendered’ health outcomes. Shift from harmful masculinities as the focus of gender outcomes.
Both outcomes and processes	9/20 assessed process, of which 8 assessed both process and outcomes. 11/20 assessed outcomes alone.
Behaviours more important to assess when assessing changes in gender outcomes	10 assessed both attitudinal and behavioural change, while 4 each assessed only one of these. (*N* = 18)
Gender transformation to be assessed at multiple levels: individual, interpersonal, community, and policy	13/20 assessed changes only at the individual level, 5/20 assessed interpersonal changes within the household, and one each assessed community-level and policy-level changes.
Emphasis on using context-specific and intersectional outcomes and indicators to assess gender transformative changes	Does not use context-specific and intersectional outcomes and indicators to assess gender transformative changes
**HOW TO ASSESS**	
Develop outcome indicators in consultation with the community	Outcomes and indicators are developed apriori
Flexibility in changing the outcomes and indicators to be assessed as the intervention unfolds	Fixed and standardised indicators used
Evaluation carried out through participatory processes with the intervention participants	Two of 20 interventions used participatory evaluation methods. In other instances, intervention participants were respondents in the evaluation
Qualitative and quantitative methods to be used; qualitative methods were most frequently mentioned.	11/20 interventions used qualitative as well as quantitative methods, 5 used qualitative methods alone, and 4 used quantitative methods alone. Of the 11 interventions using both methods 6 interventions had separate quantitative and qualitative evaluation studies, and 5 were mixed method studies.
**WHEN TO ASSESS**	
Ongoing	Wide variations in the number and timing of the evaluation studies, with most (18/20) being evaluated at 2 (10/20) or 3 (8/20) time points. One intervention was assessed only once, while another was assessed at 4 time points. The timing of the evaluations was as follows: During: 1 Baseline + End line: 6 During+ End line: 4 Baseline + End line + ≤2 years post intervention: 4 During + End line + ≤2 years post intervention: 4 During + End line + ≤2 years post intervention +6 years post intervention: 1

Consistent with interview participants’ observations, published quantitative evaluation studies used experimental, quasi-experimental, or ‘before-and-after’ study designs. The GEM scale, a tool some interview participants reported using, was also used in six evaluation studies of four interventions.

The qualitative evaluation studies also used a range of methods, including observation, public testimonies, stories of the most significant change in their lives, and a process-tracing case study of a policy change. However, participatory methods involving the communities were used only in two studies reporting on the same household-level intervention to enhance gender equality and reduce family violence against girls in humanitarian settings (Table 3) [45,46].

It is important to note that the GEM and the GRAS tools mentioned by the interview participants relied on self-reports to assess changes in attitudes toward gender equality. Moreover, 16 (of 20) interventions that aimed to change men’s gender attitudes and behaviour relied exclusively on men’s self-reports of more gender-equal attitudes and behaviours to assess the intervention’s success [18,19,22–24,26–31,35,42–44,47,48,53]. Only three interventions included reports from men’s female partners in all their evaluations [32–34,57,59]. In two interventions, some evaluation studies relied on men’s self-reports of positive changes in gender outcomes [36,51]. In contrast, in other assessments of the same interventions, men’s self-reports were corroborated by their female partners’ reports [37,38,49,50].

### When to assess/measure

Across all interview participants, there was an understanding that endline assessment alone was inadequate. Midline assessment was considered essential for course correction, and the standard practice appeared to be to conduct assessments at least three times: baseline, midline, and endpoint. In some instances, researchers or implementers returned several years after an intervention was completed to assess whether the gains in gender equality were sustained.

Something we’re very proud of regarding the (xx) programme is that we had the chance to go and do another impact assessment six years after the programme ended. And that was recent, not long ago … we (met) let’s say, a little bit more than 80 percent of the participants in the programme that was implemented between 2013 and 2015. (P22)

Two others adopted routine engagement with the community through the intervention to understand the change process, track unintended consequences, and undertake course correction at each step (P11, P12).

sort of progressive monitoring at different points. Because many times, the things happening at various points don’t seem to resurface at the endline. (P12)

Partners are gonna innovate all the time, and they are not always going to adhere to … . what you deemed the gold standard. But if you are not there regularly, you only find out at the end, and it’s too late. It might have been a good intervention. They just needed somebody there to understand what the issues were and to help them. (P11)

There was wide variation in the number and timing of the evaluations of the interventions examined. Most (18/20) were evaluated at two (10/20) or three (8/20) time points. One intervention was assessed only once, while another was evaluated at four time points.

Whereas practitioners believed that long-term assessments of gender-transformative changes were needed to understand their sustainability, only one intervention was evaluated up to 6 years after the intervention [29–31]. The remaining 19 were evaluated within 2 years of the intervention. Eleven interventions were assessed at baseline and endline, and/or within 2 years post-intervention, while 8 were assessed during, immediately post-, and/or within 2 years post-intervention (Table 3).

### How effective were the assessments?

We do not have interview data on the effectiveness of assessments. In the published evaluations, we found that in most cases, the assessment results were equivocal and could not provide guidance on whether the intervention was successful or, if not, the reasons why.

Of the latter, the single intervention that evaluated six years after its completion used quantitative methods and found that all positive gender and health outcomes were achieved at the end-line, at two years post-intervention, and remained even after six years [31] (See Table 1, column 9). Of the 19 interventions evaluated at or within 2 years post-intervention, five [27,28,35,47,48,53–55] were unsuccessful in achieving the desired gender outcomes, and one [56] was successful. In 13 interventions, the findings were mixed. Either the interventions did not sustain their initial success [16], or achieved self-reported changes in acceptance of egalitarian gender relations without a shift in male behaviour or in entrenched beliefs about masculinity and patriarchal power among many participants [18–24,32–34,36–46]. There were also instances of evaluation studies of the same intervention conducted at the same time point but using different methods, reporting contradictory findings [49–51] (Table 1, column 9).

## Discussion

This paper set out to examine approaches to assessing progress in gender-transformative SRH interventions in sub-Saharan Africa among practitioners and researchers on the one hand and in the published literature on the other. The findings reveal similarities but essential differences in the ‘What,’ ‘How,’ and ‘When’ of assessments of gender-transformative interventions (Table 3).

### What is to be assessed

One of the significant ways in which the published evidence diverges from perspectives based on practice-based knowledge is on what is to be assessed, including a focus on altering harmful or traditional masculinities with limited attention to promoting women’s empowerment, on individual and (to a much lesser extent) interpersonal levels, and the lack of attention to health system-level changes.

Furthermore, there is no indication that the outcome measures used in the intervention-evaluation studies were tailored to local contexts or intersectional inequalities, although manifestations of gender (in)equality are context-specific and vary across population subgroups.

Importantly, more than half of the interventions have assessed outcomes alone, and of those that have monitored the process, only a few provide insights into how gender-transformative changes occur in practice, how these changes influence other power relations, and any backlash that arises.

Both sets of evidence recommend using gender- and health-related outcomes to assess progress in gender-transformative SRH interventions. In addition, the published evidence used outcome indicators that we believe do not neatly fall into either category: gender or health. These are indicators of changes in health outcomes that are premised on a positive shift towards gender-equal attitudes or behaviour, which we have categorised as ‘gendered health outcomes,’ for example, increased condom use resulting from a change in male attitudes towards safer sex practices. Other studies have reported the use of such gendered health outcomes [39]. It may be worth exploring in future research and practice whether the construction and broader use of gendered health outcome indicators can help make a more accurate assessment of gender-transformative changes alongside the achievement of SRH objectives.

### How to assess

The ‘how’ of assessment in the intervention studies diverged widely from those recommended based on practice-based knowledge. In most instances, outcome indicators are set *a priori* and not modified as the intervention unfolds. There appears to be little involvement by intervention participants and other stakeholders in either identifying the outcomes to be assessed or in implementing the assessment. It is essential to note that in many of the 44 evaluation studies, the qualitative and quantitative tools used to evaluate progress toward greater gender equality relied on self-reports from intervention participants. More importantly, most interventions with men to change patriarchal attitudes and behaviours relied exclusively on men’s self-reports of more gender-equal attitudes and behaviours to assess the intervention’s success. Reliance on self-reporting is a serious limitation because it is subject to inherent biases stemming from participants’ tendency to report acceptable behaviour or attitudes [60].

### When to assess

As for the ‘when’ of assessment, while practice-based perspectives called for continuous monitoring and long-term follow-up, most intervention studies conducted end-line or post-intervention evaluations within 2 years of completing the intervention. The literature on assessing the sustainability of development interventions suggests that post-program evaluation 3–10 years after a program ends is best positioned to indicate whether the program is likely to have a sustainable impact [61]. Thus, it is not possible to predict the long-term sustainability of even the handful of reviewed interventions that have achieved short-term success.

The literature on the measurement of gender-transformative interventions within and outside the health sector largely aligns with the practice-based perspectives of interview participants and, in some tension, with the evaluation studies we reviewed. Change is to be measured across multiple levels – individual, interpersonal (within and beyond the household), and structural (e.g. laws and policies, economic structures, and health systems) [8,62]. The tools used to measure change need to be flexible, able to track non-linear, backlash, and unanticipated change [8,62], and nuanced enough to capture not only the amount and direction of change but also the depth and quality [7]. There is agreement that the processes used for monitoring, evaluation, and learning about gender-transformative change must be participatory throughout to avoid reinforcing unequal power relations [6–8] and to reflect the transformation in power relations that gender-transformative programs seek to achieve [7]. Evaluations must attempt to measure program contributions to change processes over significantly longer time frames, extending beyond the lifespan of an individual project [6].

Feminist scholars, as well as multilateral agencies engaged in implementing gender-transformative programs, have outlined the essential characteristics of gender-responsive assessment approaches suitable for interventions aimed at gender transformation, many of which were also highlighted by the interview participants. These characteristics include (a) examining the differential impact of the intervention within and across groups of women, men, and non-binary people in varied social locations (by race, ethnicity, and class, etc.), (b) tracking the intervention’s impact on the changes in power differentials (e.g. access to and control over resources and decision-making) by gender and social location, (c) ensuring that participants from less powerful groups are well-represented, and (d) prioritising their concerns in measurement tools [62–65].

To summarise, the measures and processes used by most of the reviewed intervention-evaluation studies assessing gender-transformative changes in SRH programs in sub-Saharan Africa appear to be neither fully aligned with practice-based perspectives from the region nor with the broader literature on principles for measurement and assessment in gender-transformative programs.

It is possible that the published evaluation studies we reviewed do not fully capture the measures and processes used to evaluate the interventions. Not all outcomes may have been published in journals, given limitations on article length, publication costs, and the lengthy review processes involved. However, it is such published studies that count as ‘robust evidence’ on which other interventions are funded or assessed, and it is important to understand the limitations of this body of evidence.

The use of ‘self-reported’ measures to assess successes in gender transformation by the perpetrators of inequalities; the absence of measures to assess institutional and structural changes – especially health system changes – that could foster and sustain gains in gender equality and health equity; the unequivocal findings on the short-term effectiveness of many gender-transformative SRH interventions and the lack of information on the long-term sustainability of any gains recorded, suggests that our understanding about how to design and implement ‘successful’ gender-transformative SRH interventions in sub-Saharan Africa is seriously limited.

Some of the reasons underlying this state of affairs may be gleaned from the interviews with practitioners and researchers from the region. The interviews suggest that donors’ funding for so-called ‘gender-transformative’ health interventions often provides project-based, short-term funding of two to three years without a clear commitment to continued support for both implementation and assessment. The extent of investment is limited, given the entrenched nature of gender-power inequalities and the need for multi-level interventions involving multiple actors. Although the long-term and complex nature of gender-transformative changes is well documented, organisations are pressured to demonstrate success, i.e. tangible gains in gender equality and health equity, within a two- to three-year funding cycle. These compulsions result in programs targeted at the individual level, which can lead to some changes in self-reported attitudes and, to a lesser extent, behaviour by the end of the project period.

### Strengths and limitations of the study

While there are many publications on how to assess or evaluate gender-transformative interventions, ours may be among the few studies (if any) to comprehensively examine the outcomes, processes, methodological approaches, timing, and frequency of intervention evaluations, with a specific focus on sub-Saharan Africa. The integration and comparison of evidence from practice-based knowledge and published literature are other unique features of the study. By drawing on the strengths of both approaches, the study makes a critical contribution to strengthening the assessment of gender-transformative SRH interventions in the region and in other LMIC contexts.

The study has several limitations. To begin with, the data used in this study are drawn from two independent (though related) studies. As a result, there was no one-to-one correspondence in the topics covered, and comparisons across themes are uneven. Secondly, our analysis may not have captured the full extent of the measures used to evaluate gender-transformative SRH interventions due to publication constraints and biases. Further, we only looked for published French and English reports and papers available online. We may have missed relevant literature published in other languages and not available online.

Future research should prioritise the documentation and amplification of experiential knowledge and the mobilisation of practitioners’ insights, grounded in a deeper understanding of contextual realities and power dynamics, across the design, implementation, and evaluation of gender-transformative sexual and reproductive health interventions.

## Conclusions

Transforming gender power equations is a complex, non-linear process that calls for going beyond conventional quantitative or qualitative evaluations using standardised tools at fixed time points during and immediately after a project.

Building on the strengths of practice-based perspectives and published evaluation studies helps us define the path forward. The first step involves community engagement at all stages of a gender-transformative SRH (and any) program, from setting priorities through design, implementation, and evaluation. This engagement will ensure that objectives and program design are aligned with the ‘what,’ ‘how,’ and ‘when’ of assessment, as these elements are closely connected.

Gender-transformative SRH programs must assess not only health and gender outcomes but also whether changes in health outcomes have resulted from shifts in gender-equitable attitudes and behaviours, and, importantly, in gender power relations. Wider use of ‘gendered’ health outcome indicators would help achieve this. Women’s empowerment should feature alongside shifts in men’s gender attitudes as gender outcomes to be assessed. Tools assessing shifts in gender-equitable attitudes and behaviour, when used with men, must be validated by administering them to their partners and other relevant individuals to ensure robustness of the results.

Adopting bottom-up and participatory processes for assessing gender-transformative change would enable the identification of context-specific indicators of improvements in gender equality.

Quantitative assessments of interventions must be supplemented by qualitative studies that employ participatory processes to provide insight into how the trajectories of gender-transformative change may differ across contexts and population subgroups. Flexibility in the assessment process to accommodate the exploration of new themes, mechanisms, or indicators would enable the capture of the nuances of the change process.

Evaluation studies at specific time points (e.g. baseline, midline, endline) should be accompanied by continuous engagement and documentation. When results are equivocal, further probing should be conducted to elucidate the underlying factors, to contribute to the evidence base on what works where, how, and why. And finally, it is imperative to ascertain, through evaluations conducted 3–10 years post-intervention, the sustainability of the changes brought about by any successful gender-transformative SRH program, to allow for an accurate assessment of the program’s potential.

It has been more than three decades since ‘gender-transformative approaches’ were conceptualised. The need of the hour is to invest time and money in multi-sectoral and multidisciplinary collaborations that bring together the strengths of practice-based knowledge and rigorous evidence from intervention studies to advance and operationalise nuanced, gender-responsive approaches to assessing progress in eliminating gendered barriers to poor SRH.

## Supplementary Material

PRISMA ScR Checklist.docx

## Data Availability

The authors confirm that the data supporting the findings of this study are available within the article [and/or] its supplementary materials.
